# Associations between serum soluble transferrin receptor and the prevalence of cancers

**DOI:** 10.3389/fonc.2022.1039930

**Published:** 2022-12-08

**Authors:** Yuzhuo Zhang, Nianci Xue, Wenyu Jia, Xikang Chen, Xuezhang Chen, Hongliang Li, Bin Wang, Yi Guo, Ju Chen, Huaqin Tian

**Affiliations:** ^1^ The 8th Clinical Medical College, Guangzhou University of Chinese Medicine, Foshan, China; ^2^ Foshan Hospital of Traditional Chinese Medicine, Guangzhou, China; ^3^ The Second Clinical Medical College, Guangzhou University of Chinese Medicine, Guangzhou, China

**Keywords:** biomarker, serum soluble transferrin receptor, cancer, NHANES, cross-section study

## Abstract

**Background:**

As increasing experimental evidence suggests that iron metabolism play crucial roles in cancer and non-cancer conditions, there is a lack of data on serum soluble transferrin receptor (sTfR), a promising marker representing unmet cellular iron demands, between cancer risk from epidemiological studies. Here, we aimed to evaluate the predictive value of sTfR and cancer prevalence.

**Materials and methods:**

We analyzed on 5,480 adult participants from 2015 to 2018 National Health and Nutrition Examination Survey (NHANES). Spearman correlation analysis was performed to investigate the correlations between sTfR and other characteristics. To identify the associations between sTfR and the prevalence of cancers, stratified multivariable logistic regression models, subgroup and sensitivity analyses were also performed.

**Results:**

In tertile analyses, participants in the highest level of sTfR were significantly associated with increased prevalence of total cancers [odds ratio (OR) = 1.53, 95% confidence interval (CI): 1.15-2.02] as compared with those at the lowest tertile. Each unit increment in ln-transformed sTfR concentration was shown to be associated with 39% increased risks of total cancers. Similar associations were found in males rather than females. Further subgroup and sensitivity analyses indicated that, in continuous and tertile analyses, sTfR was more closely associated with male- and female-specific cancers of prostate and testis (2.35: 1.03-5.40; 2.03: 1.00-4.09; respectively), and breast, cervix, ovary and uterus (1.92: 1.11-3.35; 1.66: 1.02-2.69; respectively).

**Conclusions:**

Our findings suggested that elevated level of sTfR was associated with the prevalence of cancers, especially in sex-specific cancers. In order to better determine them, further research in humans will be required.

## Introduction

Iron homeostasis involves in multiple cellular biological processes, including enzymatic activity, mitochondrial function and DNA synthesis (1). Unequivocally, disorders of iron metabolism, including iron deficiency and overload, and corresponding representing markers such as serum iron, ferritin, transferrin, and transferrin saturation (TSAT), closely relate with numerous cancer (2, 3). Mostly, high iron load, also called “ferrotoxic”, has been highlighted as a risk factor for cancer from basic science (4). However, evidence from several population-based prospective studies suggests mixed results on iron status and cancer risk. Some indicated that participants at higher levels of serum iron and TAST were subjected to increased risks of total cancers (5, 6), and especially breast cancer (5, 7, 8), whereas others could not deduce similar positive associations (8, 9). In addition, one recent study simultaneously analyzed the specific effects of above four markers on cancer incidence and mortality in European population, indicating that only elevated ferritin was related to lower risks of breast cancer and cancer mortality (2). Interestingly, they found that higher iron load might not contribute a cancer risk factor in the general European population.

In certain pathological conditions, such as inflammation, liver diseases and malnutrition, serum ferritin and TAST were easily affected and elevated, leading to diagnostic sophistication (10–12). Compared to them, serum soluble transferrin receptor (sTfR) seemed to be a superior and more sensitive marker representing cellular iron demands as it would not be affected by inflammation and return to normal physiological levels quickly after iron homeostasis (13, 14). TfR1 and TfR2 are two subtypes of sTfR that binds with iron-transferrin complex to facilitate serum iron into cells (15). Apart from expression on the surfaces of generic cells, TfR1 could highly express in tumor cells, resulting in higher level of sTfR (15). Thus, it attracted more attention than TfR2. Nowadays, accumulating preclinical evidence has identified that TfR1 played crucial roles in tumor onset, progression, treatment and prognosis (16–18). Notably, it is currently unknown whether these previous findings are generalizable to the results from epidemiological studies. Therefore, we report an epidemiological analysis with a large cross-sectional cohort to investigate the association between sTfR and cancer prevalence, aiming to fulfill the evidence of potential biomarkers for earlier identification of the disease.

## Materials and methods

### Study population

**Figure 1:**
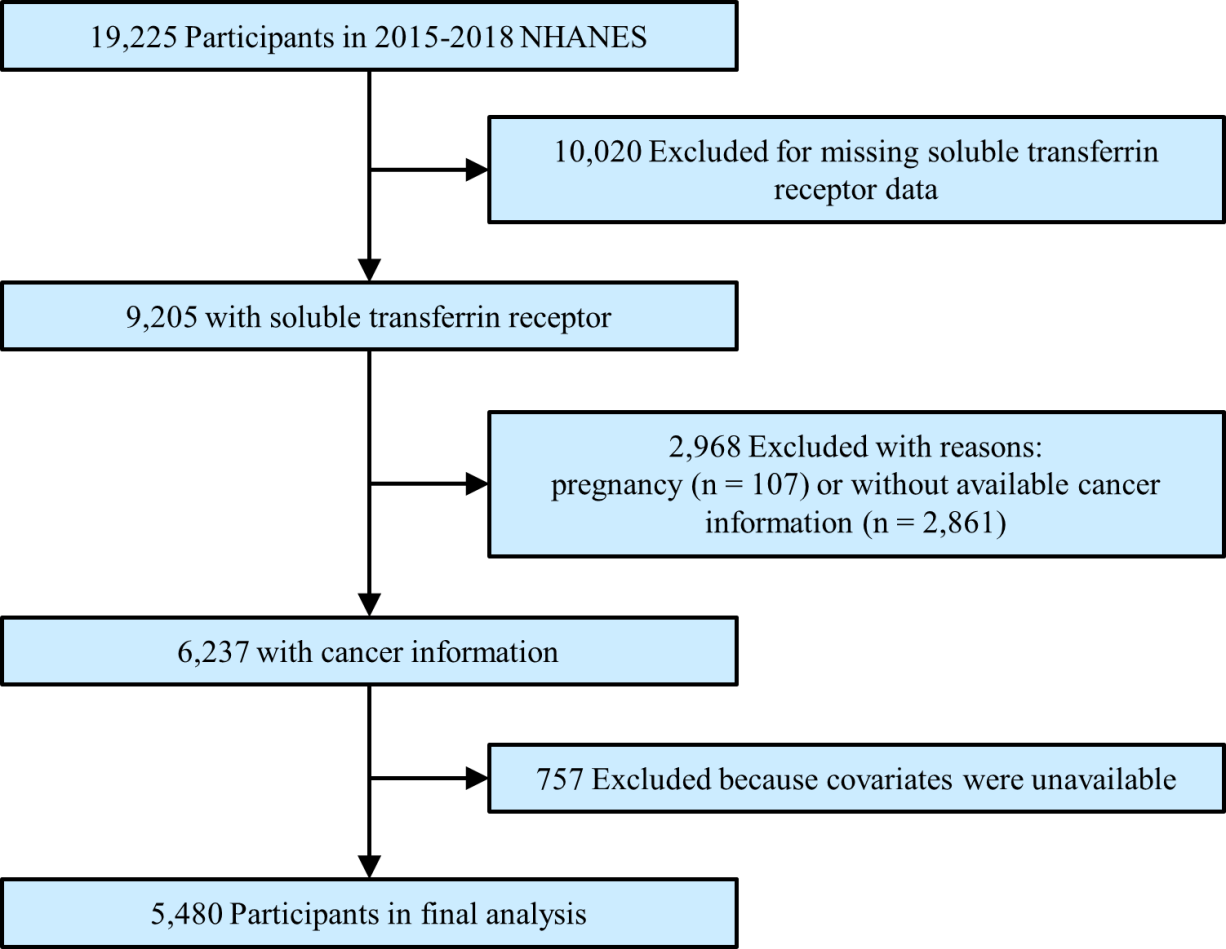
Study flowchart. Flow chart showing the process of participants selection. Of 19,225 participants from 2015 to 2018 of National Health and Nutrition Examination Survey (NHANES), 5,480 remained in the final analysis.

We enrolled participants in this study from 2015 to 2018 National Health and Nutritional Examination Survey (NHANES), a population-based national survey that focused on the health and nutrition status among US citizens. The Centers for Disease Control and Prevention ratified the study protocols, and all participants provided informed consent. All survey data and details of the operation are publicly available at www.cdc.gov/nchs/nhanes/. Our mainly interested outcomes were serum sTfR and the prevalence of cancers. Thus, the total number of participants in the primary survey was 19,225. After excluding participants missing sTfR (n = 10,020), who were pregnant (n = 107), who had unavailable cancer information (n = 2,861), or without covariate data (n = 757), a total of 5,480 subjects were enrolled in the final analysis (**Figure 1**).

### Serum soluble transferrin receptor measurement

As reported by the protocol in NHANES, serum samples were collected, processed and stored under -30°C until they were shipped to National Center for Environmental Health, Atlanta, GA for testing. The method principle for measurement of sTfR was a particle enhanced immunoturbidimetric assay that used Roche kits on the Cobas^®^ c501 clinical analyzer. The value of sTfR in mg/L was converted to nmol/L by multiplying 11.8. Other details of sTfR measurement are available at https://wwwn.cdc.gov/Nchs/Nhanes/2015-2016/TFR_I.htm.

### Assessments of total and sex-specific cancers

Total cancers were defined as a total of self-reported physician diagnoses from at least 38 kinds of malignancies, such as bladder, blood, bone, brain, colon, kidney, liver, lung, stomach, skin and lymphoma. Sex-specific cancers were classified into male- and female-specific, including one composite of prostate and testis malignancies, and another combination of breast, cervix, ovary and uterus malignancies, respectively.

### Covariate information

Following continuous and categorical covariates were included in our study, including age, body mass index, family income, systolic/diastolic blood pressure, serum iron, ferritin, hemoglobin, total protein, total cholesterol, high-density lipoprotein cholesterol, glycated hemoglobin (HbA1c), hypersensitive C-reactive protein (hs-CRP), systemic immune-inflammation index, energy intake, total fat intake, protein intake, iron intake, gender, ethnicity, education, smoking, drinking, diabetes, hypertension and cardiovascular diseases. Systemic immune-inflammation index was calculated using the peripheral blood cell counts with the calculation: (neutrophils × platelets)/lymphocytes, as it is a meaningful predictor for cancers (19). Cardiovascular diseases were defined as a composite of any self-report heart failure, coronary heart disease, myocardial infarction, angina pectoris and stroke, according to previous researches (20). The detailed acquisition process and measuring method of remaining variables are available at www.cdc.gov/nchs/nhanes.

### Statistical analysis

Baseline characteristics of all included subjects were divided into cancer-free and cancer groups, with the continuous variables reported as the median (interquartile) and the categorical variables reported as numbers with percentages. Comparisons between the two groups were performed using the χ^2^ tests (categorical variables), one-way ANOVA tests (normal distribution), or Kruskal-Wallis tests (skewed distribution). On one hand, univariate and multivariate stepwise selection regression analyses were used to explore potential related clinical factors to total cancers. On the other hand, bivariate associations between sTfR and baseline characteristics were examined using Spearman correlation analyses for continuous variables and box plots for categorical variables. Moreover, in analyses examining associations with the prevalence of cancers, sTfR was treated as continuous independent variable, scaled per 1-unit increment in ln-transformed for the skewed distribution, or divided into tertiles, using multivariable logistic regression models with different adjustments to calculate odds ratios (ORs) and corresponding 95% confidence intervals (CIs). Model I was not adjusted for any confounders, and Model II was adjusted for age, gender and ethnicity. Model III was fully adjusted for age, gender, ethnicity, body mass index, family income, education, smoking, drinking, diabetes, hypertension, cardiovascular diseases, systolic/diastolic blood pressure, serum iron, ferritin, total protein, total cholesterol, high-density lipoprotein cholesterol, hemoglobin, HbA1c, hs-CRP, systemic immune-inflammation index and nutrition intake. Model IV was only adjusted for significant difference covariate at baseline.

Additionally, several subgroup and sensitivity analyses were performed by a multivariate regression analysis. First, we conducted different subgroups, such as gender, age, ethnicity, smoking, drinking and two levels of serum ferritin and hs-CRP, to identify potential effect modifiers. To test for statistical significance of interactions, interaction terms between sTfR and different subgroups were generated and examined by the Wald test for dichotomous variables and the likelihood ratio test for multilevel variables. If necessary, the possible interactions between all adjusted factors were also tested. Second, we performed a sensitivity analysis after exclusion of patients without diagnosis of male- and female-specific cancers, including prostate, testis, breast, cervix, ovary and uterus malignancies, to investigate possible associations between sTfR and sex-specific cancers. A value of *p* < 0.05 (two-sided) was considered statistically significant. All analyses were performed with EmpowerStats software with R (version 3.4.3).

## Results

### Baseline characteristics

**Table 1** presents the baseline characteristics of a total of 5,480 participants enrolled in the study, including 498 and 4,982 with and without cancer, respectively. Among all the participants, 38.27% were males, 35.51% were Non-Hispanic Whites, and the median age at enrolment was 46 (33-62) years old. Overall, there were significant differences in baseline characteristics between the two groups, with the exception of body mass index, education, drinking, serum iron, hemoglobin, total cholesterol, high-density lipoprotein cholesterol, total fat intake and iron intake. Compared to the participants without cancer, subjects with cancer were older, smoker and had more comorbidities, such as diabetes, hypertension and cardiovascular diseases, with higher levels of systolic blood pressure, serum ferritin, sTfR and inflammation markers, such as hs-CRP and systemic immune-inflammation index.

**Table 1:** Baseline characteristics of NHANES participants included in this study^a^.

Variable	Overall (n = 5,480)	Cancer-free (n = 4,982)	Cancer (n = 498)	*P* value
Soluble transferrin receptor, nmol/L	35.50 (29.30-44.70)	35.30 (29.10-44.68)	37.60 (31.25-45.38)	<0.001
Age, years	46.00 (33.00-62.00)	44.00 (32.00-60.00)	68.00 (54.25-78.00)	<0.001
Males, (%)	2097 (38.27)	1882 (37.78)	215 (43.17)	0.018
Body mass index, kg/m^2^	28.70 (24.60-33.70)	28.70 (24.50-33.70)	28.70 (25.40-33.35)	0.572
Family income, mean income/poverty ratio	2.11 (1.18-4.00)	2.07 (1.15-3.95)	2.44 (1.33-4.47)	0.027
Ethnicity, (%)				<0.001
Non-Hispanic White	1946 (35.51)	1650 (33.12)	296 (59.44)	
Non-Hispanic Black	1218 (22.23)	1143 (22.94)	75 (15.06)	
Other	2316 (42.26)	2189 (43.94)	127 (25.50)	
Education, (%)				0.609
Lower than high school	983 (17.94)	901 (18.09)	82 (16.47)	
High school	1262 (23.03)	1149 (23.06)	113 (22.69)	
More than high school	3235 (59.03)	2932 (58.85)	303 (60.84)	
Smoking status, (%)				<0.001
Never Smoker	3312 (60.44)	3068 (61.58)	244 (49.00)	
Smoker	2168 (39.56)	1914 (38.42)	254 (51.00)	
Current drinking	1055 (19.25)	969 (19.45)	86 (17.27)	0.239
Systolic blood pressure, mmHg	122.00 (111.00-134.00)	122.00 (111.00-133.00)	127.00 (117.00-143.00)	<0.001
Diastolic blood pressure, mmHg	72.00 (65.00-79.00)	72.00 (65.00-79.00)	71.00 (64.00-77.00)	<0.001
Serum iron, μmol/L	14.30 (10.60-18.40)	14.30 (10.60-18.40)	14.30 (11.10-18.10)	0.644
Ferritin, μg/L	88.90 (40.60-173.00)	87.60 (39.00-172.00)	111.50 (63.32-190.75)	<0.001
Total protein, g/L	71.00 (69.00-74.00)	71.00 (69.00-74.00)	70.00 (67.00-73.00)	<0.001
Total cholesterol, mmol/L	4.73 (4.11-5.46)	4.73 (4.14-5.46)	4.73 (4.06-5.48)	0.656
HDL-C, mmol/L	1.33 (1.11-1.63)	1.34 (1.11-1.63)	1.32 (1.09-1.63)	0.570
Hemoglobin, g/dL	13.80 (12.90-14.80)	13.80 (12.90-14.80)	13.70 (12.80-14.60)	0.096
HbA1c, (%)	5.50 (5.20-5.90)	5.50 (5.20-5.90)	5.70 (5.40-6.20)	<0.001
hs-CRP, mg/L	2.01 (0.89-4.60)	2.01 (0.88-4.58)	2.16 (1.05-4.83)	0.013
Systemic immune-inflammation index	448.57 (316.98-629.81)	445.13 (316.86-624.95)	472.49 (318.78-692.36)	0.013
Energy intake, kcal	1911.00 (1448.75-2474.25)	1911.00 (1456.00-2490.50)	1876.00 (1409.00-2312.50)	0.004
Protein intake, gm	70.81 (52.40-95.29)	70.81 (52.66-95.82)	70.34 (50.47-89.90)	0.014
Total fat intake, gm	74.58 (53.00-101.86)	74.58 (52.85-102.34)	74.58 (53.98-96.84)	0.338
Iron intake, mg	11.89 (8.46-16.34)	11.89 (8.47-16.36)	11.89 (7.99-15.70)	0.170
Diabetes, (%)	915 (16.70)	773 (15.52)	142 (28.51)	<0.001
Hypertension, (%)	1866 (34.05)	1594 (32.00)	272 (54.62)	<0.001
Cardiovascular diseases, (%)	575 (10.49)	449 (9.01)	126 (25.30)	<0.001

HDL-C, high-density lipoprotein cholesterol; HbA1c, glycated hemoglobin; hs-CRP, hypersensitive C-reactive protein.

a Values for categorical and continuous variables with skewed distribution are expressed as n (%) and median (interquartile ranges), respectively

### Clinical factors related to total cancers

As demonstrated in **Table 2**, univariate regression analyses revealed that numerous traditional risk factors, such as age, smoking, chronic diseases, systolic blood pressure, HbA1c, and iron markers like ferritin and sTfR were positively related to increased total cancers. Accordingly, after fully adjustment with stepwise selection in multivariate regression analyses, only age, smoking, ferritin and sTfR remained significantly associated with increased risks of total cancers, in which the OR of sTfR was highest (OR: 1.39, 95% CI: 1.01-1.91, *p* = 0.0443).

**Table 2:** Analysis of clinical factors related to total cancers in univariate and multivariate regression.

Variables	Univariate regression		Multivariate regression	
	Odds ratio (95% CI)	*P* value	Odds ratio (95% CI)	*P* value
Age	1.068 (1.061, 1.074)	<0.0001	1.065 (1.056, 1.074)	<0.0001
Body mass index	0.999 (0.987, 1.012)	0.9239		
Female (Y/N)	0.799 (0.663, 0.963)	0.0183	1.235 (0.950, 1.604)	0.1143
Family income	1.098 (1.037, 1.162)	0.0012	1.059 (0.988, 1.135)	0.1042
Smoking (Y/N)	1.669 (1.387, 2.007)	<0.0001	1.286 (1.037, 1.595)	0.0221
Drinking (Y/N)	0.864 (0.678, 1.102)	0.2395	1.022 (0.771, 1.356)	0.8795
Diabetes (Y/N)	2.172 (1.762, 2.677)	<0.0001	1.229 (0.914, 1.653)	0.1724
Hypertension (Y/N)	2.558 (2.124, 3.082)	<0.0001	1.132 (0.901, 1.423)	0.2859
Cardiovascular diseases (Y/N)	3.420 (2.733, 4.278)	<0.0001	1.192 (0.913, 1.555)	0.1968
Systolic blood pressure	1.017 (1.013, 1.021)	<0.0001	0.993 (0.987, 0.999)	0.0229
Diastolic blood pressure	0.983 (0.975, 0.991)	0.0003	1.002 (0.992, 1.012)	0.7139
Energy intake^a^	0.810 (0.672, 0.976)	0.0266	1.159 (0.747, 1.800)	0.5102
Protein intake	0.996 (0.994, 0.999)	0.0040	1.001 (0.996, 1.005)	0.8054
Total fat intake	0.998 (0.996, 1.000)	0.0954		
Iron intake	0.995 (0.983, 1.007)	0.3897		
Serum iron	0.998 (0.984, 1.012)	0.7932		
Total protein	0.923 (0.903, 0.943)	<0.0001	0.985 (0.962, 1.009)	0.2166
Systemic immune-inflammation index^a^	1.176 (0.991, 1.397)	0.0635		
hs-CRP	1.009 (1.000, 1.019)	0.0580		
Hemoglobin	0.956 (0.902, 1.013)	0.1297		
Total cholesterol	1.015 (0.929, 1.108)	0.7470		
HDL-C	0.947 (0.754, 1.190)	0.6415		
Ferritin^a^	1.296 (1.187, 1.415)	<0.0001	1.138 (1.007, 1.285)	0.0379
HbA1c	1.144 (1.066, 1.228)	0.0018	0.877 (0.771, 0.997)	0.0454
Soluble transferrin receptor^a^	1.324 (1.055, 1.661)	0.0154	1.389 (1.008, 1.914)	0.0443

HDL-C, high-density lipoprotein cholesterol; HbA1c, glycated hemoglobin; hs-CRP, hypersensitive C-reactive protein.

a Values for continuous variables with skewed distribution were ln-transformed.

### Correlations of serum sTfR with baseline characteristics

**Figure 2:**
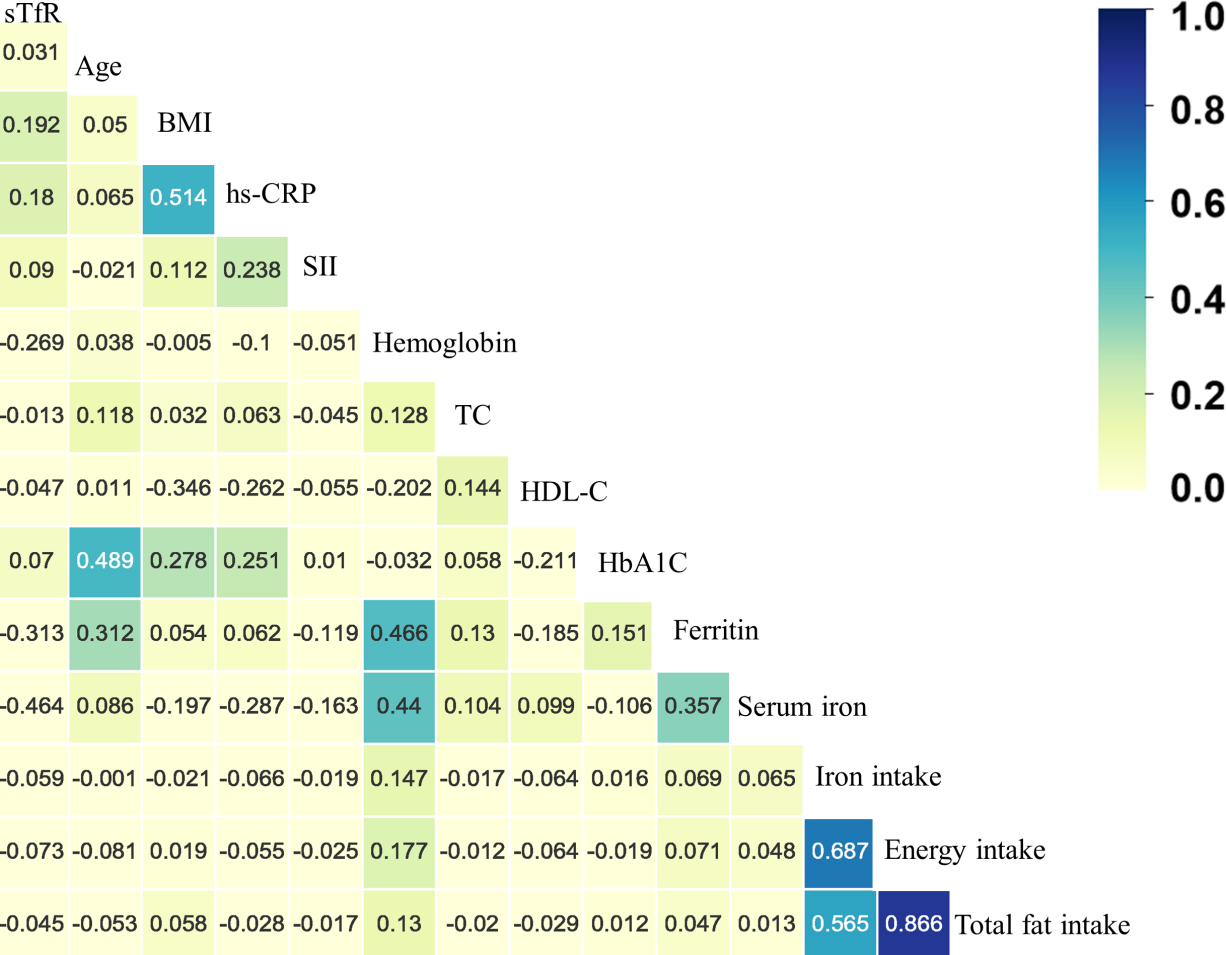
The heatmap of the correlation between covariates and serum soluble transferrin receptor (sTfR) using the Spearman correlation analysis among total participants. BMI, body mass index; hs-CRP, hypersensitive C-reaction protein; SII, systemic immune-inflammation index; TC, total cholesterol; HDL-C, high-density lipoprotein cholesterol; HbA1c, glycated hemoglobin.

In **Supplementary Figure S1** and **Table S1**, higher sTfR levels were observed in those who were female, non-Hispanic Blacks, non-smokers, non-drinkers, diabetes, hypertension, cardiovascular diseases and sex-specific cancers. Additionally, as shown in **Figure 2** and **Supplementary Tables S2**, the results of Spearman correlation analysis indicated that age, body mass index, HbA1c and inflammation markers like hs-CRP and systemic immune-inflammation index were weakly positively correlated with sTfR (all *p* < 0.05), whereas high-density lipoprotein cholesterol, hemoglobin, serum iron, ferritin, and nutrition intake were weakly negatively correlated with sTfR (all *p* < 0.01). Moreover, there was no significant correlation between total cholesterol and sTfR (*p* = 0.342).

### Associations of serum sTfR with total and sex-specific cancers

**Figure 3:**
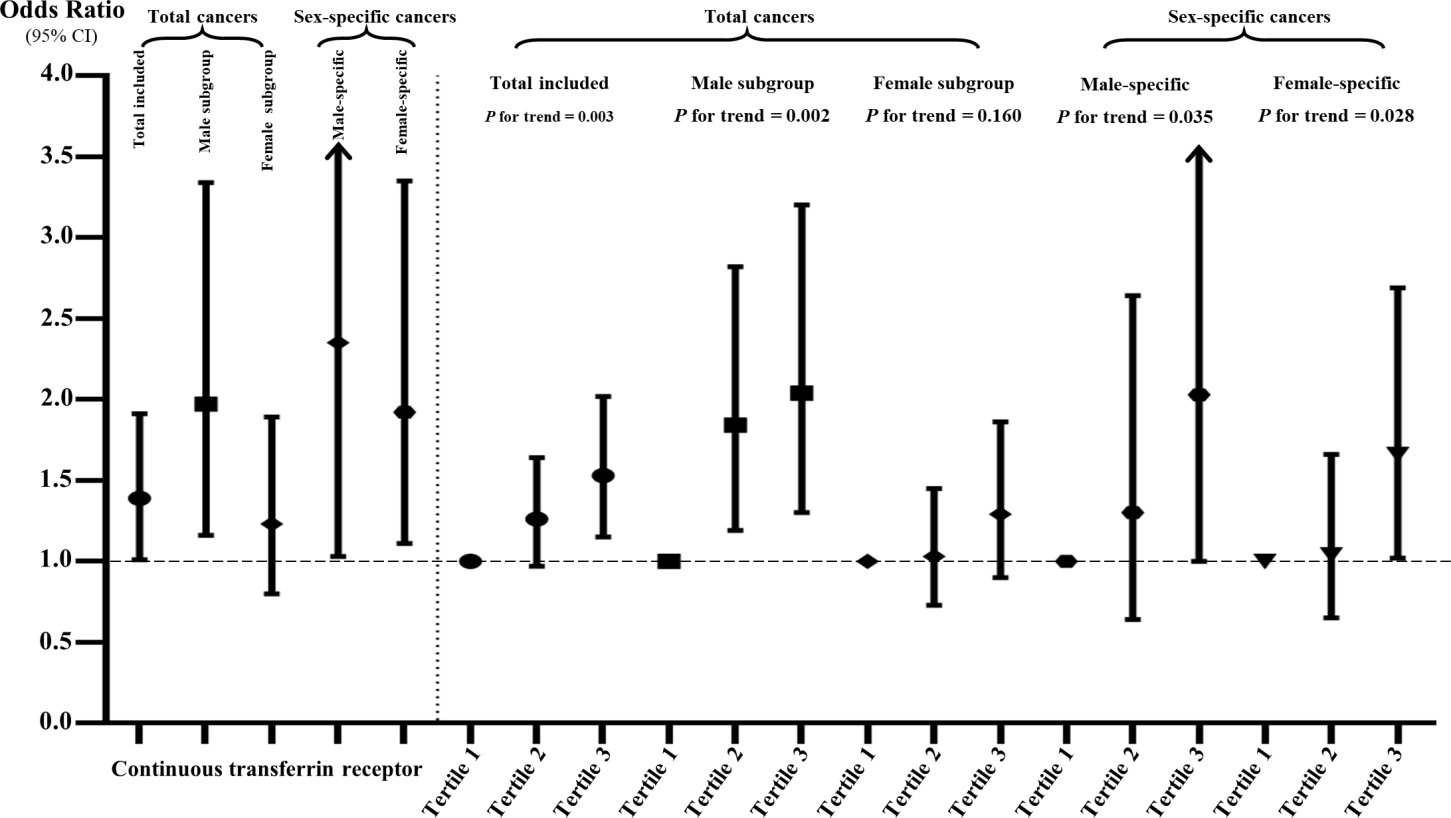
The associations of sTfR with total and sex-specific cancers in continuous and tertile analyses after full adjustment. The sTfR concentrations were ln-transformed in the continuous analysis.

**Figure 4:**
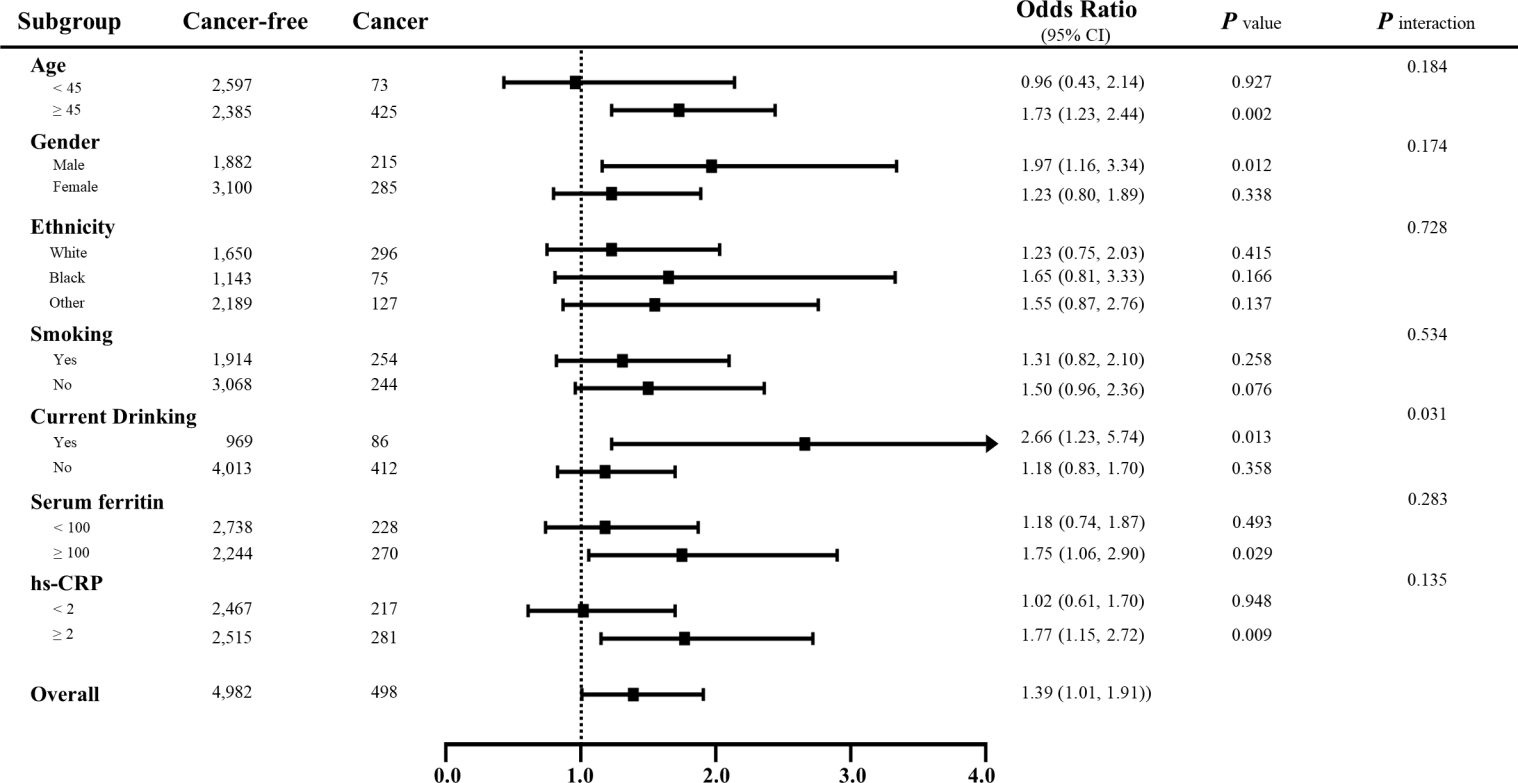
Subgroup analyses for the associations between sTfR and the prevalence of total cancers stratified by participant characteristics. Results are expressed as multivariable-adjusted odds ratio in continuous analyses after controlling covariates including age, gender, ethnicity, body mass index, family income, education, smoking, drinking, diabetes, hypertension, cardiovascular diseases, systolic/diastolic blood pressure, serum iron, ferritin, total protein, total cholesterol, high-density lipoprotein cholesterol, hemoglobin, HbA1c, hs-CRP, systemic immune-inflammation index and nutrition intake, where possible interactions between above factors are also adjusted if necessary. In the continuous analyses, the value of variables was ln-transformed.

The multivariate logistic regression results are shown in **Supplementary Table S3** and **Figure 3**. When treating serum sTfR as continuous variable, the increase in sTfR (per unit ln-transformed) was significant associated with the prevalence of total cancers in Models I to IV (OR: 1.32, 95% CI: 1.05-1.66, *p* = 0.0155; 1.53: 1.17-2.01, *p* = 0.0019; 1.39: 1.01-1.91, *p* = 0.0444; 1.53: 1.15-2.03, *p* = 0.0038; respectively). Compared to those at the lowest tertile, the prevalence of total cancers for participants in the highest level of sTfR was 1.66, 1.60, 1.53 and 1.59 in the four models, respectively. Nonetheless, after conducting a subgroup analysis by gender, for males, the positive associations of sTfR with total cancers remained obvious in continuous and tertile analysis regardless of any adjustments, which was not the case for females. Moreover, other subgroup analyses suggested that the associations between sTfR and increased prevalence of total cancers were still significant across subgroups with drinking, age over 45, higher levels of ferritin and hs-CRP (**Figure 4** and **Supplementary Table S4**). In the sensitivity analysis that excluded subjects without diagnosis of sex-specific cancers (prostate and testis malignancies for males, n = 133; breast, cervix, ovary and uterus malignancies for females, n = 138), the ORs of sTfR for male- and female-specific cancers were 2.35 (1.03-5.40, *p* = 0.0431) and 1.92 (1.11-3.35, *p* = 0.0207), respectively, in fully adjusted Model III of continuous analysis (**Figure 3** and **Supplementary Table S5**). Similarly, the positive associations were found for the top tertile of sTfR and increased prevalence of male- and female-specific cancers (2.03: 1.00-4.09, *p* = 0.0484; 1.66: 1.02-2.69, *p* = 0.0415; respectively). Furthermore, in Model IV that we only adjusted the significant difference covariates in **Table 1**, the associations between sTfR and the prevalence of sex-specific cancers were still robust in both continuous and tertile analyses.

## Discussion

In this study, out findings suggested: 1) elevated serum sTfR was associated with increased prevalence of total cancers, which was significant in males rather than females; 2) positive associations were found in both male- and female-specific cancers, including prostate, testis, breast, cervix, ovary and uterus malignancies; 3) serum sTfR was positively correlated to age, body mass index, HbA1c and inflammation while inversely correlated to HDL-C, serum iron, ferritin and iron intake. To our knowledge, this is the first study with large sample size to assess the associations between sTfR and the prevalence of cancers. More importantly, multivariate regression analyses with stepwise selection indicated the closest association between sTfR and total cancers compared to other common clinical tumorigenesis factors, and numerous crucial potential confounders such as comorbidities, lifestyles, nutrition intake, hs-CRP, systemic immune-inflammation index and previously reported iron status markers, have been adjusted in present study. Consistent with a previous study that revealed a potential sex dimorphism between cancer mortality and iron status (2), we did observe the significant association between sTfR and the prevalence of total cancers among men, but not the women. Probably, it was attributed to the inhibitory effect of estrogen and estrogen receptor 1 on TfR1 (21, 22). Traditionally, hormones could decrease with aging and the age of 45 to 50 was considered as a watershed threshold, which was more prevalent and fluctuated in female. Also, the larger proportion of female in the subgroup with age over 45 might account for the higher sTfR levels in total female participants. Likewise, it seems to be the evidence supporting the association between higher sTfR and increased total cancer prevalence in this subgroup. Collectively, age might affect sTfR expression and then lead to tumorigenesis. Besides, it is intriguing that when we only analyzed the sex-specific cancers, there was a positive value of sTfR to predict cancer prevalence among both men and women. On one hand, further separate analyses on type-specific cancer prevalence were restricted considering the limited number of individual cancer type. On the other hand, the interaction between gender, age and the detailed mechanism underlying the predictive value of sTfR in cancer merit further investigation.

There are many preclinical studies of iron overload and increased cancer risks. One of the most prominent was that iron stimulated hydroxyl radical formation, leading to oxidative tissue damage and subsequent carcinogenesis (23). Alternatively, iron could promote tumor growth through hypoxia-inducible factor (HIF) and WNT pathways (4). However, the results of Spearman correlation analysis in present study demonstrated inversed associations with sTfR of iron intake, serum iron and ferritin, which mean that higher levels of iron status might not always be a risk factor for cancer prevalence to some extent. A further line of evidence on inversely effect of ferritin between cancers comes from meta-analysis (24). Possible proposed mechanism underling such inverse associations was that iron deficiency could affect immune function and DNA biosynthesis (25, 26). In sight of this, we referred ferritin < 12 μg/l as anemia (prevalence of 5.88% in present study) and found it not associate increased total cancer risks. It is worth noting that elevated sTfR patients who were drinking or exposed in higher level of hs-CRP, had increased risks of total cancer prevalence. But interestingly, non-drinkers and even non-smokers in this study had higher sTfR levels instead. One previous study summarized that alcohol could alter the levels of some iron-related proteins, including serum iron, hepcidin and transferrin, which was not the case for sTfR (27). It indicated that there might be a more complex indirect interaction between alcohol consumption, sTfR and tumorigenesis. Currently, researches on the relationship between tobacco and sTfR always focused on newborns (28). Thus, further research on this topic in adults is meaningful in the future. Obviously, the burden of alcohol/tobacco-attributable cancers was explicit and highlighted endlessly. Hence, encouraging lifestyle improvements to the resolution of chronic inflammation remains an essential element for cancer prevention.

Inevitably, our study has some limitation. First, the nature of observational epidemiological study prevents us to draw any causal relationship between sTfR and cancer prevalence. Comparatively, serum sTfR as a potential earlier warning marker can be established. Second, the subtypes of sTfR were unavailable from NHANES raw data, which indicated that both TfR1 and TfR2 might be included in the study. Third, we tested all associations with single sTfR measurement but the fluctuation of individual sTfR levels was not available in our study, which restricted further investigation of the time-course associations between changes in sTfR and the incidence of new cancer events. Finally, on one hand, we only had sufficient statistical power for investigations on the prevalence of total and sex-specific cancers as the limited number of individual type-specific cancer. On other hand, some subgroups with a small number of events might exhibit a potentially statistical bias. Thus, the results should be interpreted with caution in clinical practice.

## Conclusion

Overall, in this large cohort, we suggest that sTfR may be a potential earlier warning marker for the prevalence of total cancers, and particularly in sex-specific cancers. Obviously, further evidence on these potential links between sTfR and cancer risk from well-designed human studies should be warranted.

## Data availability statement

The original contributions presented in the study are included in the article/**Supplementary Material**. Further inquiries can be directed to the corresponding authors.

## Ethics statement

This study was approved by the ethics review board of the National Center for Health Statistics and written informed consents were obtained from each participant. The patients/participants provided their written informed consent to participate in this study.

## Author contributions

YZ, NX, WJ, XKC, XZC, HL, BW,YG, JC, and HT conceived and designed the study. YZ and NX collected and analyzed the data. YZ drafted the paper and HT revised the manuscript. All authors contributed to the article and approved the submitted version.
