# Supplementary material for: Associations between serum soluble transferrin receptor and the prevalence of cancers
Source: Front Oncol. 2022 Dec 8;12:1039930. doi: 10.3389/fonc.2022.1039930 (PMC9773974; doi:10.3389/fonc.2022.1039930)
Supplement: Supplementary file 1 [file DataSheet_1.docx]

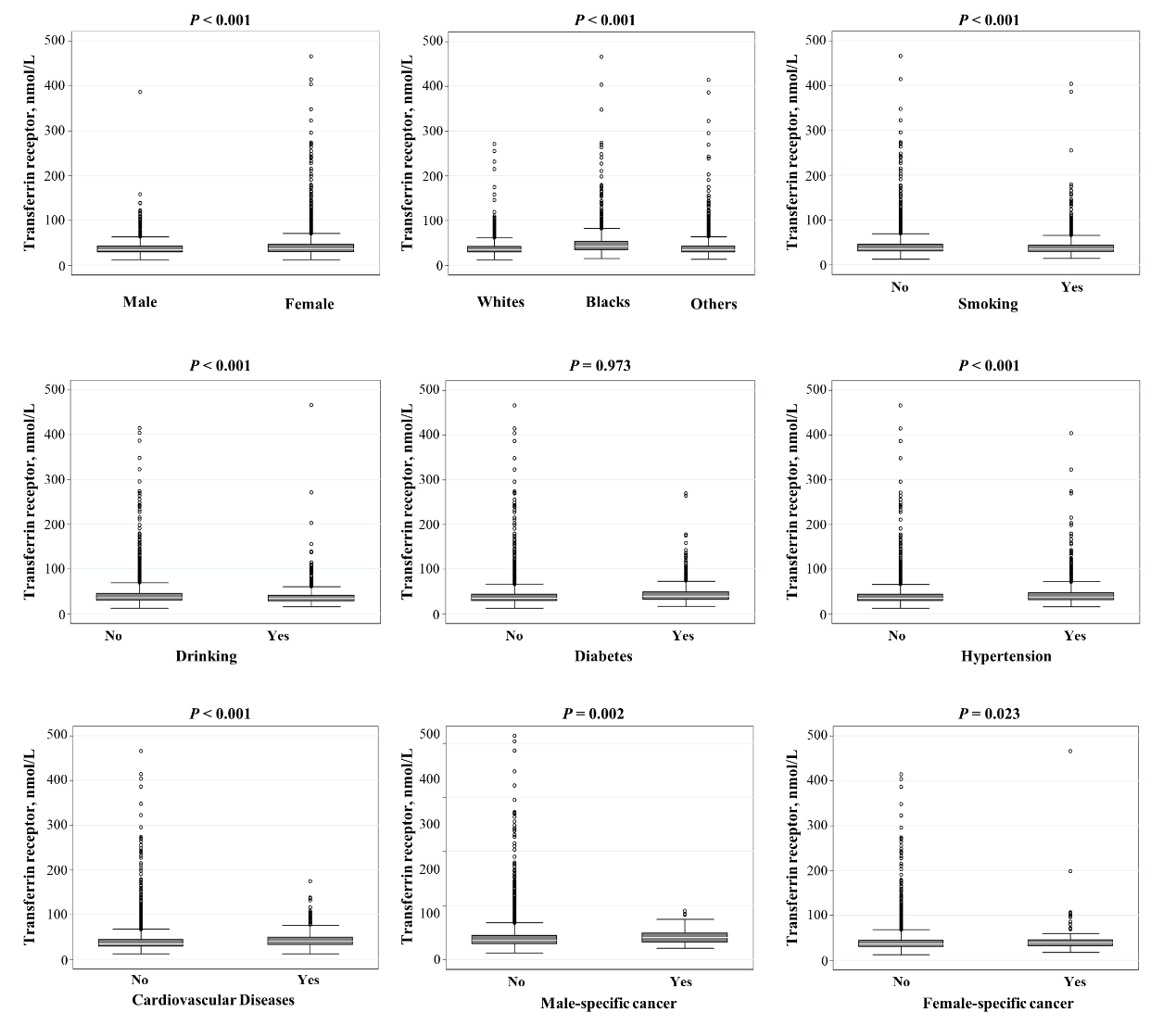
Figure S1. Soluble transferrin receptor concentrations by categorical participant characteristics and prespecified outcomes. Box plots for transferrin receptor for sex, race/ethnicity, smoking status, current smoking status, diabetes, hypertension, cardiovascular diseases and sex-specific cancers. The middle white line of the box represents the median value with the edges of the box representing the 25th and 75th percentiles. The whiskers extend to 1.5× (interquartile range), and the dots represent values falling outside that range. *P* *value* determined by Kruskal–Wallis tests that treat continuous transferrin receptor concentrations as the dependent variable and the participant characteristic as the independent variable.

| Table S1. Soluble transferrin receptor concentrations by baseline categorical variables and prespecified outcomes | | | |  |
| --- | --- | --- | --- | --- |
|  |  |  |  |  |
|  | Mean (SD) | Median (IQR) | *P* value |  |
| Sex |  |  | <0.001 |  |
| Male | 37.5 (16.0) | 34.1 (28.4-42.4) |  |  |
| Female | 43.2 (28.4) | 36.5 (29.9-46.3) |  |  |
| Race |  |  | <0.001 |  |
| Non-Hispanic White | 37.7 (17.0) | 34.0 (28.6-41.9) |  |  |
| Non-Hispanic Black | 49.5 (33.3) | 41.4 (34.0-53.1) |  |  |
| Other | 39.3 (23.6) | 34.3 (28.3-42.5) |  |  |
| Smoking Status |  |  | <0.001 |  |
| Never | 42.1 (26.4) | 35.9 (29.7-45.5) |  |  |
| Ever & Current | 39.4 (21.3) | 34.9 (28.6-43.4) |  |  |
| Current Drinking |  |  | <0.001 |  |
| No | 41.8 (25.1) | 36.0 (29.7-45.5) |  |  |
| Yes | 38.0 (22.2) | 33.6 (27.8-40.9) |  |  |
| Diabetes |  |  | <0.001 |  |
| No | 40.5 (25.0) | 34.9 (28.9-43.9) |  |  |
| Yes | 43.7 (22.4) | 38.9 (31.3-48.2) |  |  |
| Hypertension |  |  | <0.001 |  |
| No | 40.3 (24.9) | 34.7 (28.7-43.5) |  |  |
| Yes | 42.5 (24.0) | 37.3 (30.4-46.8) |  |  |
| Cardiovascular diseases |  |  | <0.001 |  |
| No | 40.7 (25.2) | 35.0 (29.0-44.1) |  |  |
| Yes | 43.8 (18.7) | 40.1 (32.0-49.3) |  |  |
| Male-specific cancer |  |  | 0.002 |  |
| No | 40.9 (24.4) | 35.3 (29.1-44.7) |  |  |
| Yes | 43.1 (14.8) | 40.9 (32.2-49.2) |  |  |
| Female-specific cancer |  |  | 0.023 |  |
| No | 40.9 (24.4) | 35.3 (29.1-44.7) |  |  |
| Yes | 45.0 (40.7) | 38.8 (31.2-45.2) |  |  |
| SD, standard deviation; IQR, interquartile range. | | | |  |

| Table S2 *P* values of the Spearman correlation analysis between baseline variables and soluble transferrin receptor among total participants | | | | | | | | | | | | | | |  |
| --- | --- | --- | --- | --- | --- | --- | --- | --- | --- | --- | --- | --- | --- | --- | --- |
| Variable | sTfR | Age | BMI | hs-CRP | SII | Hemoglobin | TC | HDL-C | HbA1c | Ferritin | Serum iron | Iron intake | Energy intake | Total fat intake |  |
| sTfR | 0.000 | 0.022 | 0.000 | 0.000 | 0.000 | 0.000 | 0.342 | 0.001 | 0.000 | 0.000 | 0.000 | 0.000 | 0.000 | 0.001 |  |
| Age | 0.022 | 0.000 | 0.000 | 0.000 | 0.119 | 0.005 | 0.000 | 0.418 | 0.000 | 0.000 | 0.000 | 0.970 | 0.000 | 0.000 |  |
| BMI | 0.000 | 0.000 | 0.000 | 0.000 | 0.000 | 0.716 | 0.016 | 0.000 | 0.000 | 0.000 | 0.000 | 0.125 | 0.155 | 0.000 |  |
| hs-CRP | 0.000 | 0.000 | 0.000 | 0.000 | 0.000 | 0.000 | 0.000 | 0.000 | 0.000 | 0.000 | 0.000 | 0.000 | 0.000 | 0.041 |  |
| SII | 0.000 | 0.119 | 0.000 | 0.000 | 0.000 | 0.000 | 0.001 | 0.000 | 0.446 | 0.000 | 0.000 | 0.162 | 0.064 | 0.216 |  |
| Hemoglobin | 0.000 | 0.005 | 0.716 | 0.000 | 0.000 | 0.000 | 0.000 | 0.000 | 0.017 | 0.000 | 0.000 | 0.000 | 0.000 | 0.000 |  |
| TC | 0.342 | 0.000 | 0.016 | 0.000 | 0.001 | 0.000 | 0.000 | 0.000 | 0.000 | 0.000 | 0.000 | 0.199 | 0.359 | 0.139 |  |
| HDL-C | 0.001 | 0.418 | 0.000 | 0.000 | 0.000 | 0.000 | 0.000 | 0.000 | 0.000 | 0.000 | 0.000 | 0.000 | 0.000 | 0.034 |  |
| HbA1c | 0.000 | 0.000 | 0.000 | 0.000 | 0.446 | 0.017 | 0.000 | 0.000 | 0.000 | 0.000 | 0.000 | 0.238 | 0.168 | 0.390 |  |
| Ferritin | 0.000 | 0.000 | 0.000 | 0.000 | 0.000 | 0.000 | 0.000 | 0.000 | 0.000 | 0.000 | 0.000 | 0.000 | 0.000 | 0.000 |  |
| Serum iron | 0.000 | 0.000 | 0.000 | 0.000 | 0.000 | 0.000 | 0.000 | 0.000 | 0.000 | 0.000 | 0.000 | 0.000 | 0.000 | 0.333 |  |
| Iron intake | 0.000 | 0.970 | 0.125 | 0.000 | 0.162 | 0.000 | 0.199 | 0.000 | 0.238 | 0.000 | 0.000 | 0.000 | 0.000 | 0.000 |  |
| Energy intake | 0.000 | 0.000 | 0.155 | 0.000 | 0.064 | 0.000 | 0.359 | 0.000 | 0.168 | 0.000 | 0.000 | 0.000 | 0.000 | 0.000 |  |
| Total fat intake | 0.001 | 0.000 | 0.000 | 0.041 | 0.216 | 0.000 | 0.139 | 0.034 | 0.390 | 0.000 | 0.333 | 0.000 | 0.000 | 0.000 |  |
| sTfR, soluble transferrin receptor; BMI, body mass index; hs-CRP, hypersensitive C-reactive protein; SII, systemic immune-inflammation index; TC, total cholesterol; HDL-C, high-density lipoprotein cholesterol; HbA1c, glycated hemoglobin. | | | | | | | | | | | | | | |  |
|  |  |  |  |  |  |  |  |  |  |  |  |  |  |  |  |

| Table S3 Associations between soluble transferrin receptor and total cancers in continuous and categorial analysis | | | | |
| --- | --- | --- | --- | --- |
|  | Odds ratio (95% CI), *P* value | | |  |
|  | Model I^a^ | Model II^b^ | Model III^c^ | Model IV^d^ |
| Total participants (n = 5,480) | |  |  |  |
| Continuous sTfR^e^ | **1.32 (1.05, 1.66) 0.0155** | **1.53 (1.17, 2.01) 0.0019** | **1.39 (1.01, 1.91) 0.0444** | **1.53 (1.15, 2.03) 0.0038** |
| Categories |  |  |  |  |
| Tertile 1 | Reference | Reference | Reference | Reference |
| Tertile 2 | **1.42 (1.12, 1.80) 0.0042** | 1.25 (0.97, 1.61) 0.0889 | 1.26 (0.97, 1.64) 0.0860 | 1.27 (0.98, 1.64) 0.0747 |
| Tertile 3 | **1.66 (1.32, 2.10) <0.0001** | **1.60 (1.24, 2.06) 0.0003** | **1.53 (1.15, 2.02) 0.0030** | **1.59 (1.22, 2.08) 0.0006** |
| P for trend | <0.001 | <0.001 | 0.003 | <0.001 |
| Males (n = 2,097) |  |  |  |  |
| Continuous sTfR^e^ | **2.93 (1.96, 4.37) <0.0001** | **2.14 (1.36, 3.37) 0.0011** | **1.97 (1.16, 3.34) 0.0118** | **2.22 (1.36, 3.62) 0.0014** |
| Categories |  |  |  |  |
| Tertile 1 | Reference | Reference | Reference | Reference |
| Tertile 2 | **1.78 (1.22, 2.60) 0.0027** | **1.69 (1.12, 2.54) 0.0118** | **1.84 (1.19, 2.82) 0.0057** | **1.82 (1.20, 2.78) 0.0053** |
| Tertile 3 | **2.79 (1.93, 4.03) <0.0001** | **2.10 (1.40, 3.15) 0.0003** | **2.04 (1.30, 3.20) 0.0019** | **2.17 (1.41, 3.35) 0.0004** |
| P for trend | <0.001 | <0.001 | 0.002 | <0.001 |
| Females (n = 3,383) |  |  |  |  |
| Continuous sTFR^e^ | 0.95 (0.71, 1.29) 0.7602 | 1.17 (0.83, 1.66) 0.3695 | 1.23 (0.80, 1.89) 0.3377 | 1.24 (0.86, 1.79) 0.2508 |
| Categories |  |  |  |  |
| Tertile 1 | Reference | Reference | Reference | Reference |
| Tertile 2 | 1.21 (0.89, 1.66) 0.2232 | 1.02 (0.73, 1.41) 0.9234 | 1.03 (0.73, 1.45) 0.8603 | 1.04 (0.75, 1.45) 0.8108 |
| Tertile 3 | 1.20 (0.88, 1.62) 0.2516 | 1.25 (0.90, 1.73) 0.1842 | 1.29 (0.90, 1.86) 0.1683 | 1.30 (0.92, 1.82) 0.1321 |
| P for trend | 0.273 | 0.169 | 0.160 | 0.122 |
| CI, confidence interval; sTfR, soluble transferrin receptor.  ^a^Crude model;  ^b^Adjusted for age, sex and ethnicity;  ^c^Adjusted for age, sex, body mass index, ethnicity, family income, education, smoking, drinking, diabetes, hypertension, cardiovascular diseases, systolic blood pressure, diastolic blood pressure, serum iron, ferritin, total protein, total cholesterol, high-density lipoprotein cholesterol, hemoglobin, HbA1c, hs-CRP, systemic immune-inflammation index, iron intake, total fat intake, energy intake, protein intake.  ^d^Only adjusted for significant factors of baselines in Table 1.  ^e^The value of variable was ln-transformed in a continuous analysis. | | | | |
|  |  |  |  |  |
|  |  |  |  |  |
|  |  |  |  |  |
|  |  |  |  |  |

| Table S4 Subgroup analyses for the associations between soluble transferrin receptor and total cancer stratified by participant characteristics in continuous analysis^a^ | | |  |
| --- | --- | --- | --- |
|  |  |  |  |
|  | Fully adjusted odds ratio (95% CI), *P* value | *P* interaction |  |
| Age |  | 0.184 |  |
| ≤ 45 (n = 2,670) | 0.96 (0.43, 2.14) 0.9268 |  |  |
| > 45 (n = 2,810) | **1.73 (1.23, 2.44) 0.0018** |  |  |
| Gender |  | 0.174 |  |
| Males (n = 2,097) | **1.97 (1.16, 3.34) 0.0118** |  |  |
| Females (n = 3,383) | 1.23 (0.80, 1.89) 0.3377 |  |  |
| Ethnicity |  | 0.728 |  |
| Non-Hispanic White (n = 1,946) | 1.23 (0.75, 2.03) 0.4149 |  |  |
| Non-Hispanic Black (n = 1,218) | 1.65 (0.81, 3.33) 0.1658 |  |  |
| Other (n = 2,316) | 1.55 (0.87, 2.76) 0.1368 |  |  |
| Smoking status |  | 0.534 |  |
| Smoker (n = 2,168) | 1.31 (0.82, 2.10) 0.2577 |  |  |
| Never Smoker (n = 3,312) | 1.50 (0.96, 2.36) 0.0760 |  |  |
| Current drinking |  | 0.031 |  |
| Yes (n = 1,055) | **2.66 (1.23, 5.74) 0.0131** |  |  |
| No (n = 4,425) | 1.18 (0.83, 1.70) 0.3583 |  |  |
| hs-CRP |  | 0.135 |  |
| < 2 (n = 2,684) | 1.02 (0.61, 1.70) 0.9482 |  |  |
| ≥ 2 (n = 2,796) | **1.77 (1.15, 2.72) 0.0089** |  |  |
| Overall | 1.39 (1.01, 1.91) 0.0444 |  |  |
| CI, confidence interval; hs-CRP, hypersensitive C-Reactive protein.  Fully adjusted for age, body mass index, ethnicity, family income, education, smoking, drinking, diabetes, hypertension, cardiovascular diseases, systolic blood pressure, diastolic blood pressure, serum iron, ferritin, total protein, total cholesterol, high-density lipoprotein cholesterol, hemoglobin, HbA1c, hs-CRP, systemic immune-inflammation index, iron intake, total fat intake, energy intake, protein intake.  ^a^The value of variable was ln-transformed in a continuous analysis. | | |  |
|  |  |  |  |
|  |  |  |  |
|  |  |  |  |
|  |  |  |  |

| Table S5 Associations between soluble transferrin receptor and sex-specific cancers in continuous and categorial analysis | | | | |
| --- | --- | --- | --- | --- |
|  | Odds ratio (95% CI), *P* value | | |  |
|  | Model I^a^ | Model II^b^ | Model III^c^ | Model IV^d^ |
| Male-specific (n = 1,964) | |  |  |  |
| Continuous sTfR^e^ | **3.56 (1.98, 6.40) <0.0001** | **1.98 (1.02, 3.85) 0.0443** | **2.35 (1.03, 5.40) 0.0431** | **2.26 (1.08, 4.71) 0.0304** |
| Categories |  |  |  |  |
| Tertile 1 | Reference | Reference | Reference | Reference |
| Tertile 2 | 1.46 (0.78, 2.72) 0.2390 | 1.27 (0.66, 2.46) 0.4749 | 1.30 (0.64, 2.64) 0.4719 | 1.35 (0.68, 2.69) 0.3969 |
| Tertile 3 | **3.32 (1.88, 5.84) <0.0001** | **1.93 (1.05, 3.56) 0.0348** | **2.03 (1.00, 4.09) 0.0484** | **2.09 (1.08, 4.05) 0.0290** |
| P for trend | <0.001 | 0.029 | 0.035 | 0.022 |
| Female-specific (n = 3,245) | |  |  |  |
| Continuous sTfR^e^ | 1.14 (0.77, 1.68) 0.5149 | 1.50 (0.97, 2.31) 0.0681 | **1.92 (1.11, 3.35) 0.0207** | **1.62 (1.02, 2.55) 0.0389** |
| Categories |  |  |  |  |
| Tertile 1 | Reference | Reference | Reference | Reference |
| Tertile 2 | 1.15 (0.74, 1.79) 0.5218 | 1.02 (0.65, 1.60) 0.9357 | 1.04 (0.65, 1.66) 0.8637 | 1.05 (0.67, 1.66) 0.8273 |
| Tertile 3 | 1.37 (0.90, 2.07) 0.1382 | 1.51 (0.98, 2.33) 0.0612 | **1.66 (1.02, 2.69) 0.0415** | **1.61 (1.03, 2.51) 0.0375** |
| P for trend | 0.133 | 0.050 | 0.028 | 0.026 |
| CI, confidence interval; sTfR, soluble transferrin receptor.  ^a^Crude model;  ^b^Adjusted for age and ethnicity;  ^c^Adjusted for age, body mass index, ethnicity, family income, education, smoking, drinking, diabetes, hypertension, cardiovascular diseases, systolic blood pressure, diastolic blood pressure, serum iron, ferritin, total protein, total cholesterol, high-density lipoprotein cholesterol, hemoglobin, HbA1c, hs-CRP, systemic immune-inflammation index, iron intake, total fat intake, energy intake, protein intake.  ^d^Only adjusted for significant factors of baselines in Table 1.  ^e^The value of variable was ln-transformed in a continuous analysis. | | | | |
|  |  |  |  |  |
|  |  |  |  |  |
|  |  |  |  |  |
|  |  |  |  |  |
